# The Search as Learning Spaceship: Toward a Comprehensive Model of Psychological and Technological Facets of Search as Learning

**DOI:** 10.3389/fpsyg.2022.827748

**Published:** 2022-03-15

**Authors:** Johannes von Hoyer, Anett Hoppe, Yvonne Kammerer, Christian Otto, Georg Pardi, Markus Rokicki, Ran Yu, Stefan Dietze, Ralph Ewerth, Peter Holtz

**Affiliations:** ^1^Knowledge Construction/Multimodal Interaction, IWM – Leibniz-Institut für Wissensmedien, Tübingen, Germany; ^2^Visual Analytics, TIB – Leibniz Information Centre for Science and Technology, Hannover, Germany; ^3^L3S Research Center, Leibniz University Hannover, Hannover, Germany; ^4^Information Design, Hochschule der Medien, Stuttgart, Germany; ^5^Data Science and Intelligent Systems, University of Bonn, Bonn, Germany; ^6^Data & Knowledge Engineering, Heinrich-Heine-University Düsseldorf, Düsseldorf, Germany; ^7^Knowledge Technologies for the Social Sciences, GESIS – Leibniz Institute for the Social Sciences, Cologne, Germany

**Keywords:** human–computer interaction, web search, search as learning, ranking algorithms, knowledge gain, search engine interface

## Abstract

Using a Web search engine is one of today’s most frequent activities. Exploratory search activities which are carried out in order to gain knowledge are conceptualized and denoted as *Search as Learning* (SAL). In this paper, we introduce a novel framework model which incorporates the perspective of both psychology and computer science to describe the search as learning process by reviewing recent literature. The main entities of the model are the *learner* who is surrounded by a specific learning *context*, the *interface* that mediates between the learner and the information environment, the *information retrieval (IR) backend* which manages the processes between the interface and the set of Web resources, that is, the *collective Web knowledge* represented in resources of different modalities. At first, we provide an overview of the current state of the art with regard to the five main entities of our model, before we outline areas of future research to improve our understanding of search as learning processes.

## Introduction

Using an online search engine is one of today’s most frequent activities. According to Turner and Rainie (2020), 81 percent of Americans rely on information from the Internet “a lot” when making important decisions. Most web search activities do not merely consist of simply looking up a specific piece of information, such as how to get to the next supermarket; web search is most often complex and exploratory in nature (Marchionini and White, 2007). Such online search activities, as opposed to simple fact-finding or navigational tasks, are typically open-ended (Wildemuth and Freund, 2012) and aimed at sense-making and understanding of the information retrieved (Vakkari, 2016). To emphasize the learning aspect of exploratory search with the intent of understanding, potentially leading to knowledge gain, we use the term *search as learning* (SAL).

In formal learning activities, for example, consider a course unit in higher education, the learning success depends primarily on characteristics of the learner, but also on instructional design of learning resources and the quality of instruction of educators (e.g., Schneider and Preckel, 2017). In SAL activities on the other hand learners find themselves in an open, ever-growing digital information environment, which they have to navigate successfully in order to learn without any or only with limited instruction. An online resource for learning can be every website a user encounters, which means that most resources retrieved will not be informed by instructional design principles to aid learning. In addition, today’s search engines are typically not optimized for learning, but rather for the maximization of sold advertisements, relevance in terms of a document’s similarity to the query, or popularity of documents, and rank results accordingly (e.g., Machado et al., 2020). Consequently, they can present misleading or biased results when learners use them to acquire knowledge (Segev, 2010); for example, recommender systems that present to the user mostly content that is, for example, regarding political orientation similar to previously consumed content could facilitate the formation of “filter bubbles” (Pariser, 2011; Geschke et al., 2019). Other works argue that topical relevance is not a good concept to evaluate retrieval systems and suggest measuring the usefulness or utility of retrieval and ranking results (Harter, 1992; Azzopardi et al., 2018; Vakkari et al., 2019). The collective Web knowledge is represented in various resources, from which the information retrieval (IR) backend selects relevant content, contains an incalculable number of websites with varying degrees of usefulness for learning and with different modalities such as text, image, and sound. Since most of those potential learning resources were not explicitly designed for this purpose, a learner has to regulate the search as learning processes herself (Brand-Gruwel et al., 2009). This stresses the significance of applying the study of human learning to the science of information search to generate ideas how to improve the IR backend in order to support the learner.

While classical information retrieval models focus on systems and technologies (Hiemstra, 2009), already three decades ago models began to adapt a more user-centered view (Belkin, 1980; Bates, 1989; David, 1989; Kuhlthau, 1993) according to which information seekers actively progress through different stages of search behavior. Recent models emphasized the cognitive aspects of information retrieval (Vakkari, 2001; Sharit et al., 2008; Rouet and Britt, 2011), or even specified a computational model of the learner (Fu and Pirolli, 2007). Inspired by self-regulation research, those and other models further posited that learners need to constantly regulate their search behavior (Brand-Gruwel et al., 2009). There is no one-size-fits-all IR model in computer science research but instead a wide variety of models which stress different aspects of the search as learning process (Xie, 2010).

To our knowledge, there is no IR-model that provides an interdisciplinary perspective, incorporating both psychological and pedagogical research on SAL activities on the one hand, and computer and information science research—in particular, information retrieval research—on the other hand. However, both facets of SAL activities are deeply intertwined. We propose our so-called “Spaceship model” as a novel framework in order to describe relevant research insights from the fields of psychology, education, and information retrieval that contribute to the understanding of SAL activities. Previous works on characterizing SAL processes (Vakkari, 2016) largely focus on textual learning resources and corresponding theories of learning (Hoppe et al., 2018; Machado et al., 2020). This, however, does not mirror the multimedia richness of the Web and preferences of the users. Consequently, we also discuss mechanisms for multimedia information retrieval. To illustrate the key factors relevant in a SAL activity, please consider our description of the learner Louise who wants to know how thunderstorms and lightning form.

### A Vignette: Louise, the Learner

Louise (a learner) gets a homework assignment to search the Internet for information on how thunderstorms and lightning form and to summarize the information she acquired from the websites she found in the form of a brief essay.

Louise is 14 years old and has some basic knowledge on electricity and physics from school. Of course, she also knows thunderstorms and lightning from her personal experience. She somehow remembers from a children’s TV show that lightning may have something to do with friction between clouds. Louise is a bright kid and she usually prefers watching instructional videos above reading long and complicated texts. She is an average Internet user with little technical knowledge. Louise is quite motivated to hand in a decent essay, but she does not want to spend more than 2 h on this task.

Louise opens her Web browser and enters the terms “lighting” and “thunderstorms” into the query box of her favorite search engine. The Web browser accesses the IR backend and uses different features, e.g., textual similarity, to find potentially relevant indexed websites. Based on word frequency statistics, the IR backend assumes that “lighting” should indeed be “lightning” and automatically corrects the supposed mistake. Finally, Louise receives a ranked list of websites. First, some popular videos are shown, followed by more text-based resources.

Louise clicks the link to the first video. She quickly realizes that this is not what she was looking for because the video just shows video recordings of thunderstorms with dramatic music. Louise now enters the terms “lightning,” “thunderstorm,” and “friction” into the query field. The IR backend now presents a different set of results to Louise. Among them are several websites on which the formation of thunderstorms and lightning is explained for children and adolescents. Louise opens the first two links to instructional videos in separate tabs and watches the videos carefully while taking notes. From the following two links, which are mostly text-based, Louise skips the first and clicks on the second because the website’s name sounds familiar to her. Then, Louise decides that she now has enough information for her essay and terminates the Web search. The IR system retains the interaction information from Louise’s Web search session, using it in her profile.

### The “Spaceship Model”: An Overview

In the following sections, we discuss the different components of our “Spaceship model.” It was developed by reviewing IR-models (see introduction) and models of self-regulated learning (SRL; see learner sub section). Specifically, we used the stratified model of information retrieval interaction (Saracevic, 1997) as a starting point. Saracevic (1997) was insightful in developing a model containing multiple instances (strata) on the side of the user and of the computer. Those aspects made it suitable for our theoretical framework which is targeted at providing a big picture of SAL. We expand theoretical work of Saracevic (1997) by providing more detail on the individual levels involved on the user (learner) and computer (interface, IR-backend) sides in order to take an interdisciplinary approach combining information science and psychology.

**Figure 1:**
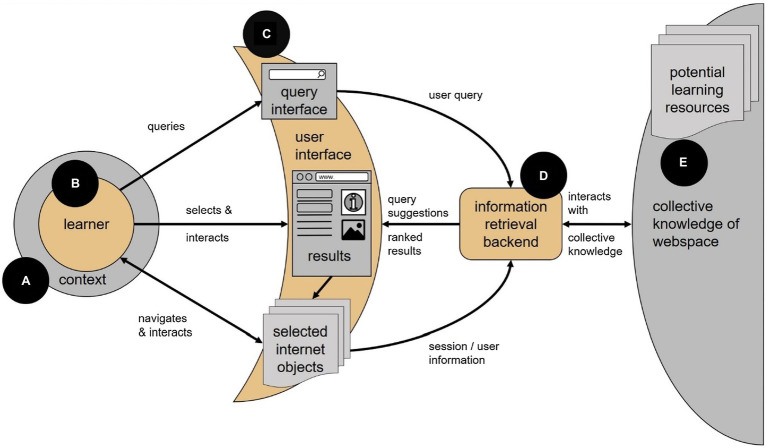
Search as Learning “Spaceship” framework model with all its components.

The main entities within our spaceship model are (A) the learner’s *context* [Section “Context (A)”], (B) the *learner* [Section “Learner (B)”], (C) the *interface* [Sections “User Interface (C)” and “Learner and Interface”] that mediates between the learner and the information environment, (D) the *IR backend* [Section “Information Retrieval Backend (D)”] which manages and mediates the processes between the interface and set of Web resources, that is (E) the *collective knowledge of Web resources* [Section “Web Resources (D)”] representing potentially all available knowledge resources in the Web (see Figure 1). Finally, the long-term dynamics of SAL processes are discussed in Section “Long-Term Dynamics.”

Similar to most other IR models, the Spaceship model can also be read as a process model representing the temporal succession of (most often iterative) learning activities of the user and processing steps within the technical environment. Initially, SAL activities are triggered by voluntary learning goals or extraneously imposed learning tasks that can be part of a formal educational learning context. In both cases, the cognitive system monitors and regulates interface interactions and learning activities (Flavell, 1979; Zimmerman and Schunk, 2001) until a learning goal has been met or another stop-rule has been triggered (Brand-Gruwel et al., 2009).

## Components

### Context (A)

Although researchers often emphasize the importance of context when it comes to information search behavior, most often, no clear definition is provided (Agarwal, 2017). For our framework, we consider context as the container in which the phenomenon of interest (the learner and his/her learning processes) resides (Dervin, 2003). The learner’s goals, actions, and even motives are influenced by the context (Salili et al., 2001; Volet, 2001). We can illustrate the influence of the context by looking at Louise (see Section “A Vignette: Louise, the Learner”). Her SAL activity has been triggered by an extraneous source of motivation, namely homework. Louise does not face a time limit; she is free to search and learn in a self-regulated manner over an extended period of time and several search sessions. She can stop her learning process at any time she likes. Since Louise is at home, she can use a laptop instead of the tablet provided at school. The browser at home, installed at her personal computer, is another part of the context in which she is moving. Her personal information saved within her set-up, for example, through cookies or a permanent login into a Google-account, can lead to a personalized search context with results based on her user profile [see Section “Information Retrieval Backend (D)”] Beyond that, personal contextual boundary conditions of motivation, time, location, device, and regulation, Louise is also challenged by a specific type of learning task. She is not just facing a simple fact-finding (or look-up) search with a clearly defined end (closed-end task) but needs to reach the higher learning goal of synthesizing the information that retrieved into something new (i.e., the essay; cf. Anderson and Krathwohl, 2001) which is connected to the task complexity.

Therefore, in our model, the task and the linked complexity can be seen as another important factor of the context influencing Louise. With increasing task complexity, the complexity of domain information, problem solving information, and the number of sources increases (Byström and Järvelin, 1995). This interplay, between task complexity and the search for information (Vakkari, 1999), affects learners’ search process (Walhout et al., 2017). Although the perceived task complexity can change for learners while working on a task (Liu et al., 2011), monitoring and assessing the complexity helps to understand the SAL process. For example, Ghosh et al. (2018) observed that with increasing task complexity (ranging from understanding to being able to evaluate strategy/method), the produced search queries increased in length. Walhout et al. (2017) compared three search tasks differing in complexity (fact-finding, understanding cause-effect chain, and elaborating a complex topic). They found that more complex tasks led to more queries; it took learners longer to formulate queries and influenced the consideration of search results on the search engine result page.

Coming back to Louise, to achieve her goal of learning, Louise has to acquire both factual knowledge about single concepts (e.g., clouds, humidity, and electricity) as well as knowledge about the interactions and causalities between them. Such causal conceptual knowledge (van Genuchten et al., 2012) is well presentable through different multimodal representation formats like videos, animations, flowcharts, or pictures. Therefore, Louise may choose videos over text-based websites that are recommended to her by the search engine. Depending on the type of knowledge, the represented modalities during a search can differ. Would Louise, for example, face homework related to cognitive procedural knowledge, like learning how to calculate the volume of an octagon, she would most likely encounter fewer animations or pictures in the learning material.

### Learner (B)

**Figure 2:**
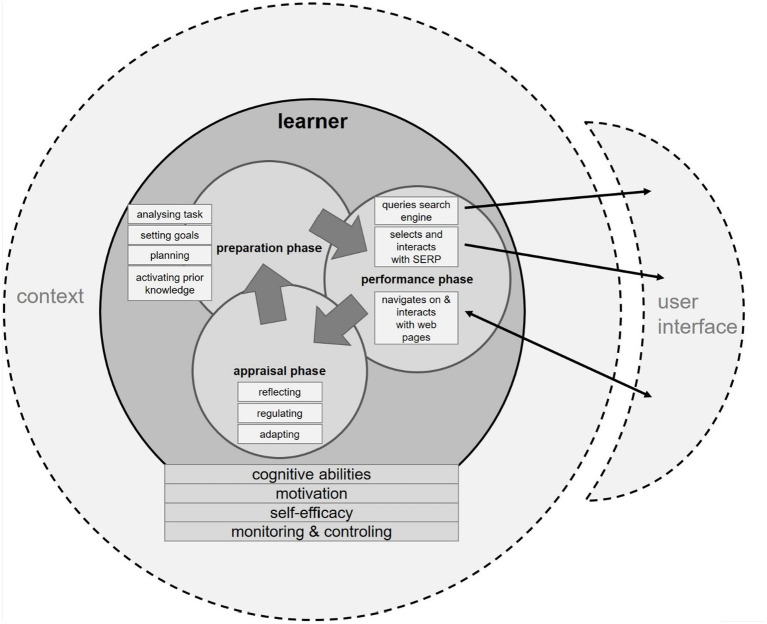
Learner submodel including the context and connections to the user interface.

Given the differences between a SAL setting and a “traditional” formal learning context, the role of the learner is pivotal for SAL activities. Consequently, in our framework, the learner and his abilities and personal characteristics are represented in a submodel of its own (Figure 2).

In the previous section, we described how the context could influence searching as learning. Learners such as Louise bring individual levels of cognitive abilities, motivation, self-regulation abilities, and prior knowledge with them. These individual factors heavily influence the learning processes in general and the SAL process as such. In the following paragraph, we discuss how learner characteristics influence SAL processes.

#### SAL as Self-Regulated Learning

Navigating in a hypermedia environment requires active control of the learner (Scheiter and Gerjets, 2007). Hence, using a search engine to learn calls for adequate self-regulation (Winne, 2001; Brand-Gruwel et al., 2009). As described in the monitoring-and-control model by Nelson (1996), learners monitor their learning behavior during SRL in order to control it. Monitoring also incorporates the assessment of one’s emerging knowledge. A variety of SRL models have been proposed since then (Panadero, 2017). We focus on those SRL aspects which are featured in all these models, namely the three main phases that are progressed cyclically (see Figure 2) during a SAL activity: *preparation*, *performance*, and *appraisal*. During preparation, learners activate their prior knowledge, set a learning goal, and activate self-motivating beliefs as well as learning strategies. Think of Louise, who remembers having watched a TV show about how lightning forms. Her learning goal is influenced by the learning context—in this case, the task description of the homework assignment. Louise is motivated to hand in a decent essay, but she does not want to spend more than two hours on this task, so she sets a certain standard of understanding the topic. As her first learning strategy, she plans to use general terms for querying the Google search engine. In the performance phase of SAL, Louise carries out her search strategy. She scans information retrieved by her query and selects a resource from the search results page (SERP) to elaborate on its content. She then processes the information of the selected resource.

Exerting cognitive control to stay engaged is important, since disengagement from the SAL task might result in poor learning performance. Louise could use metacognitive strategies like goal shielding, which inhibits distracting stimuli (Shah et al., 2002). Since a SAL activity provides multiple potential learning resources which have to be integrated (List and Alexander, 2017), Louise also needs to engage in multiple text comprehension (MTC). Because information retrieved from the Internet is likely presented in a multimodal format, using multimedia resources properly (e.g., integrating information of picture into information from text) is yet another activity which needs monitoring and regulation (Mayer, 2005). Finally, in the appraisal phase Louise evaluates her task performance and compares her learning progress to her learning goal. Here metacognitive judgments of knowledge play a crucial role (Bjork et al., 2013). Cognitive and emotional reactions are generated, which influence the start of the next SRL cycle.

The self-regulation of learning behavior in online settings can be very challenging (Bol and Garner, 2011; Whitelock-Wainwright et al., 2020). Learners typically fail to use critical SRL strategies (Azevedo, 2005) such as note taking, although those would, in most cases enhance online learning performance (Kauffman et al., 2011). One specific difficulty consists in the requirement to distribute limited cognitive resources not only to processing the information retrieved but also to the hypermedia environment itself which usually contains information that is not structured in an optimal way (Schraw, 2007). Using search engines to retrieve information also presents a challenge for the accuracy of assessing one’s own knowledge since there is evidence that using search engines to answer knowledge questions can lead to an overestimation of what is known (Pieschl, 2009; Fisher et al., 2015). Additionally, short SAL activities can lead to false certainty, where answers to knowledge test questions that were answered incorrectly are regarded as more correct than prior to a SAL activity (von Hoyer et al., 2019). Although search as learning can be challenging, digital technology can also be used as a metacognitive tool to facilitate learning (Feyzi-Behnagh et al., 2014). By incorporating theoretical assumptions on SRL in the Spaceship model, we aim to help researchers identify ways to improve human monitoring and control processes during self-regulated learning in SAL settings.

#### Motivation

Since SAL activities are cases of SRL, learners need to energize the initiation of a SAL activity somehow. As described within the goal-setting theory of motivation, individuals engage in goal-directed behavior to attain a future valued outcome (Locke and Latham, 2006). Higher task performance is usually positively related to the strength of a goal because it motivates a higher level of cognitive effort and self-regulation. Take Louise, who is motivated to “hand in a decent essay.” Her learning goal could be classified as ranging somewhere between high and low. Since she searches for completing a homework assignment it may be the case that her SAL-behavior is energized by a “have-to motivation.” If she would be interested in the topic of lightning or curios about clouds, Louise might adopt a “want-to motivation.” This kind of intrinsic goal-orientation is far superior in stimulating self-control than the previous goal orientation (Werner and Milyavskaya, 2019). Holding a high intrinsically motivated goal enables a person to regulate its learning behavior toward gaining more knowledge. This is achieved by recruiting a range of cognitive and metacognitive strategies to monitor one’s learning progress, focus the attention to the task at hand, activate prior knowledge, etc (Pintrich, 1999). In a SAL-context specific strategies targeted at the hypermedia environment have to be deployed as discussed in the previous paragraph.

Apart from learning goals, an individual’s self-efficacy—that is, the belief about one’s ability to perform a task within a specific domain—contributes to learning success (Bandura and Ramachaudran, 1994). Learners gather information about their self-efficacy from their past learning experience. Since Louise remembers some information about lightning from a TV-show, she should have the self-motivating belief that she is able to process information about the topic successfully. In general, high self-efficacy is associated positively with the use of self-regulatory strategies which promotes overall learning success (Semmar, 2006). Next to this generally type, specific internet self-efficacy is also a predictor of search task performance (Joo et al., 2000). This means Louise’s self-perception as an average Internet user should inform her motivating beliefs about her ability to search the internet for information, hence influencing her performance. In a SAL context, however, our self-efficacy might also be biased. Since google presents us with all the knowledge at our fingertips (Sparrow et al., 2011), some research points toward the possibility that experiencing the ease and speed of online information search might lead to the increased appraisal of one’s cognitive abilities (Ward, 2013). This could in turn be responsible for effects of overestimating what is knows after online information search (Fisher et al., 2015; Pieschl, 2021).

In summary, setting high learning goals energized by a want-to rather a have-to motivation accompanied by high self-efficacy for the task at hand and also high internet self-efficacy should stimulate more cognitive effort and self-control which in turn leads to greater learning performance. Research in the field of SAL should control for these motivational factors, since they are not directly observable but lie within a learner and impact not only search behavior but also learning outcome. Additionally, the hypermedia context might bias self-efficacy.

Another angle for the study of SAL could be to use motivation as dependent variable for investigating ways to support it from the side of the IR-system. From a psychological perspective, a SAL-setting holds the potential for increasing a learner’s motivation because of its hypermedia environment. Learners are free in exploring the internet and therefore experience a high degree of autonomy and control, which are factors contributing to engagement in goal-oriented behavior (Ryan and Deci, 2017). One major challenge for the learner in SAL is however, to direct attention to learning goals and not to entertaining but distracting content of the internet and engage in online procrastination (Thatcher et al., 2008). So one direction for research in SAL could be to investigate how, for example, a user interface (UI) should be designed to support a learner’s motivation and direct the attention to web content, which is helpful in reaching the learning goal and shield attention against distracting stimuli.

#### Prior Knowledge

As shown in the learner submodel (see Figure 2), prior knowledge is already activated during the preparation phase. It is not just crucial for the learning outcome itself; it also affects the behavior during SAL processes (White et al., 2009). For example, by entering queries, the learner is not just informing the IR backend about her information need but also implicitly providing information about her knowledge state. Learners with higher prior knowledge tend to formulate more topic-relevant queries for complex tasks (Monchaux et al., 2015), while learners with low prior knowledge are bad at selecting and modifying search queries which lead to relevant Web resources (Wildemuth, 2004). Furthermore, Sanchiz et al. (2017) found that prior domain knowledge helped users produce semantically more domain-specific keywords to compose queries. Monitoring the search queries and their evolution helps generate a more precise reflection of learners’ domain knowledge.

Hence, it is possible to assess learners’ prior knowledge by analyzing query complexity and the evolution of queries submitted (Yu et al., 2018). Another implicit measure of prior knowledge in SAL can be the search activity itself. For instance, learners with more prior knowledge tend to be more efficient in navigation regarding search time and the number of visited relevant web pages (Hölscher and Strube, 2000).

#### Cognitive Abilities

Learners also differ regarding their cognitive prerequisites. We want to focus exemplarily on working memory (WM) capacity (Baddeley, 2007) and reading comprehension skills (Coiro, 2011) of learners and their influences on SAL processes. We choose these two abilities as examples since they have been shown to influence in general various learning outcomes, such as academic achievement (Savolainen et al., 2008; Alloway and Alloway, 2010).

Louise is described as a child with above-average intelligence. The construct of reading comprehension describes a learner’s ability to collect information out of a text and to create a coherent mental model of the text’s content (Kintsch, 1998). With the evolution of information resources from printed offline text to digital resources, text-based resources often include (multimodal) information channels such as images or videos (Coiro, 2011). Higher reading comprehension abilities are associated with navigation strategies used during the SAL process: Learners with high reading comprehension skills are more likely to follow links with a high semantic relationship to already read content (Salmerón and Garcia, 2011).

Similarly, the concept of WM affects SAL processes as well. As defined by Baddeley (2007), WM is a cognitive system that learners use to process and manipulate information. Louise from our example is a bright kid who is able to quickly process, link, and understand new information which points toward a good WM capacity. Several studies found effects of WM capacity on SAL processes. For example, Shah et al. (2002) could demonstrate a positive relationship between the number of distinct websites visited and WM. Learners with higher working memory were also found to be able to write more comprehensive texts after a SAL session (Choi et al., 2019). Pardi et al. (2020) found that students with higher reading comprehension and WM capacity achieved overall better results when learning online about the formation of clouds and thunderstorms.

### User Interface (C)

User interfaces represent the point of contact between the user and an interactive system. They enable the IR system to receive input from and present output to the user; they enable interaction between the user and the system. In today’s IR systems, queries can be formulated in the form of keywords (consider Louise typing “thunderstorm”) or as natural language queries. Apart from typing queries by using a keyboard, modern search systems can also offer alternative forms of query entries or queries such as speech (Chang et al., 2013), drawing sketches (Liu et al., 2017), or humming melodies (Salamon et al., 2013). In this paper, we focus on keyword-based search as it is still the most pervasive form in today’s systems. In addition, Louise can interact with the search engine by clicking on query suggestions and search results that are presented by the IR system as a response to her submitted queries.

Through the user interface, the IR system reacts to the queries entered by Louise in various ways. First, most IR systems provide query auto-completion already while typing (i.e., type-ahead) as well as propose additional terms for query refinement. After the first submission of selected search query terms, the IR system further assists by spelling correction (“Did you mean.?”), related searches performed by other users (e.g., “Other users searched for.”), or semantically similar searches (e.g., “searches related to.”; see also section “Query Processing”).

Louise, however, does not seem to consider these suggestions, although she later refines the query herself by entering new query terms. As a second (though, of course, parallel) reaction to the entered or refined query terms, the search engine presents a ranked list of search results, that is, the SERP. For desktop searches, in today’s search engines, search results are typically presented on multiple consecutive SERPs between which the user can navigate back and forth (*via* navigation links presented in the footer). In contrast, for mobile searches, e.g., Google’s mobile search, the search results are presented as one endless list with infinite scrolling.

The search results of the world’s most used search engine—Google search—are ranked according to their relevance to the entered query, as well as according to many additional factors that are part of the search algorithm of an IR system, such as the popularity and the up-to-dateness of the information source (Hingoro and Nawaz, 2021). Each search result usually consists of a hyperlink that refers to the actual information resource, a title, and a brief excerpt or preview of the information resource. The title or the URL can also provide cues regarding the information provider, that is, the name or type of source that has published the information (cf. e.g., Lewandowski and Kammerer, 2021). In a SAL context, all search results, or more specifically, the information resources they link to, serve as potential learning resources. In Louise’s case, the first few search results on the SERP are vertical results linking to popular videos. Below, several organic search results that link to text-based resources are presented. However, SERPs of today’s search engines often comprise several additional elements (Azzopardi et al., 2018), such as video results, image results, or news results. Those are also called vertical results and originally stem from specialized search engines but are integrated into the main SERP. The easy access to these additional information resources provides richer and more varied opportunities for learning, but also increases the risk for the learner to get cognitively overloaded or to lose track of their actual learning goal.

Search engine result pages often include direct answers, presented at the top of the retrieved results (at position zero). They provide a direct, written (or numeric) answer to a user’s question, sometimes accompanied by an image. The presentation of direct answers might result in the fact that users do not access any information sources, as they might be satisfied with the directly provided answer (cf. Lewandowski and Kammerer, 2021). Thus, in the case of learning-oriented Web searches, this might bear the risk of too short and shallow learning episodes because learners might stop their learning already on the SERP.

Finally, it should be noted that even though today’s SERPs comprise a variety of different features, most of the individual elements are organized in a list-like manner. They thus provide strong affordance to start at the top of the list and predominantly attend to and select those elements presented first. Alternative interface designs for the exploration of search results that might reduce the strong focus on the top results are discussed by Kammerer and Gerjets (2011) and Wilson (2011). For instance, search results interfaces that group or categorize information resources according to particular types of information, such as, whether the provided information is written from a neutral and objective point of view or rather opinion-based (e.g., Kammerer and Gerjets, 2012) might support learners in the selection of appropriate information resources. Furthermore, presenting search results in a graphical overview that indicates content-related relationships between the information resources (comparable to a mind map) might foster integration across different documents (e.g., Salmerón et al., 2010). Finally, search results interfaces that indicate whether a content is disputed by other information resources (Ennals et al., 2010; Yamamoto, 2017) might increase the awareness of contradictions between information resources.

### Learner and Interface

The following section describes the interactions between the learner and the interface in a temporarily ordered process model, thereby complementing the more static description of the involved instances above.

During search, there are various possible interactions between a learner and the information retrieval system (see section “SAL as Self-Regulated Learning”). We can identify the three main activities of querying, reviewing the SERP and selecting resources, and interacting with those resources (see Figure 1). The following subsections explore interaction behaviors for each of these activities and discuss how they have been qualified, measured, and used in current SAL research. There is a number of models that aim to structure the user interaction happening during an online search. One instance is the IPS-I model (Brand-Gruwel et al., 2009) which divides the user activities contributing to search into five steps. In the following passage, we will focus on three steps (out of those five) which happen in direct interaction with the information retrieval system: (1) The search intent is defined by targeted queries; (2) The search results are inspected for suitable information sources; and (3) Chosen information sources are scanned and read.

A SAL activity is by no means a linear, three-step process. Rather, it is usually an iterative process that goes back and forth between the three activities of the performance phase and other SRL phases. Section “Long-Term Dynamics” further reflects how today’s search systems are a result of long-term interactions of users and retrieval systems.

A comprehensive framework for describing and evaluating the different steps of task-based information-interactions between a user (in our case a learner) and an information system had already been proposed previously by Ingwersen, Järvelin, and colleagues (e.g., Ingwersen and Järvelin, 2005; Järvelin et al., 2015). Our model expands these previous approaches by describing in much more details the psychological aspects as well as interactions between the interface and the information retrieval backend such as advanced crawling techniques. Nevertheless, models such as those proposed by Ingwersen, Järvelin, and colleagues can be integrated into spaceship model very well as close-ups of the central aspect of user-technology-interaction.

#### Querying

A first entry point for analysis are *queries composed by the user*. A prominent feature in SAL literature is the query length (Dommes et al., 2011; Chevalier et al., 2015; Monchaux et al., 2015; Sanchiz et al., 2017; Walhout et al., 2017). Beyond the number of words in a query, the *choice of words* has been investigated, exploring the complexity of the used language or keywords and the domain specificity (McCrudden and Schraw, 2007; Brand-Gruwel et al., 2009; Monchaux et al., 2015). Both features have been identified as indicators of a user’s prior domain knowledge. Information literate searchers tend to refer to a set of keywords for searching, while less experienced users such as children may use natural language queries to phrase their information need (Kammerer and Bohnacker, 2012; Bilal and Gwizdka, 2018). Finally, the use of specialized language, such as Boolean operators, indicates expert users, for instance, librarians or patent professionals (Dinet et al., 2004; Kim et al., 2011).

More extensive studies underline the iterative nature of search processes and allow more than one search query, and thus, iterative *query reformulation* (Rieh and Xie, 2006; Collins-Thompson et al., 2016). Studies analyzed typical reformulation behavior (e.g., Wildemuth, 2004; Jansen et al., 2007; Liu et al., 2010; Hu et al., 2013; Tibau et al., 2018, 2019; Wildemuth et al., 2018).

There is additional, implicit information communicated to the search engine by the users’ machine. Most commonly, this includes the user agent (used operating systems and browser, their versions, and the device type); the users’ locations (derived from IP address or GPS functionalities of modern devices), extended sensor data, and possible deductions about the users’ environment (e.g., in a university library, on a commute).

Modern search interfaces seek to support the users in query formulation and searching—but, as a consequence, also influence the search process (see Section “Query Processing” for details).

#### Inspection of Search Results

Interaction features can indicate what part of the search results was considered relevant—such as the SERP items which have been clicked (e.g., Buscher et al., 2012). Using several tabs to open multiple interesting Web resources for “lateral reading” has been linked to domain knowledge and search expertise. In some instances, studies also included examination of the behaviors which lead to the choice of specific SERP items. Examples for this are the measurement of the *time users spent on the SERP* (Wineburg and McGrew, 2019), detailed assessments of the users’ *scrolling behaviors* (Buscher et al., 2012; Liu et al., 2017), and eye-tracking measurements (Buscher et al., 2012; for a recent overview, see Lewandowski and Kammerer, 2021). In such studies, more time spent inspecting SERP items was, for instance, correlated with greater prior domain knowledge (Wineburg and McGrew, 2019). Other, more invasive study protocols record think-aloud protocols to access the users’ thoughts during search (Bortoluzzi and Marenzi, 2017; Ghenai et al., 2020).

Again, the design of modern search systems influences how users search. Current search engines occasionally offer additional functionalities such as the option to search for certain media types or to further limit the search space by faceted search (Diriye et al., 2010). Studies suggest that usage of facets increases with task complexity (Niu and Hemminger, 2015); their impact has been investigated for exploratory search (Kules et al., 2009), but not yet specifically for learning during search.

#### Interaction With Selected Web Resources

Even though a resource has been chosen from the SERP, its relevance for the learning task has still not been fully assessed. Consequently, the user might still decide to abandon it to inspect another resource (and then come back to it) or discard it altogether. User-centered studies examine how fast an individual user takes this decision and what information is used as a base—the site’s content or meta-data such as the author, publication date, or the perceived reliability of the source.

Observed *user behaviors within a resource* include the time and attention which is dedicated to the visible parts of the resource. Closely related is the scrolling behavior (e.g., used by Claypool et al., 2001; Liu et al., 2017), which determines which parts of a web resource are visible and thus gives indications how fast certain parts are skimmed. Some studies use eye-tracking for more detailed insights in user behavior: Eye movement measurements may indicate user interest, motivation, resource relevance, and currently used processing strategy (scanning resources vs. deep processing); users skipping irrelevant/easy/known passages points to prior domain knowledge (Alemdag and Cagiltay, 2018). Features other than those related to the users’ behavior, such as related to the Web resources and their content, are discussed in Section “Web Resources (D).”

Finally, users may discover additional resources during their reading process, by either following HTML links in the consulted Web resources or by following indications in the resources’ semantic content (e.g., if another author is referenced without providing an explicit Web link).

#### Abandoning Search

The user can abandon the search at any point, that is, they can decide to stop search and terminate the learning process or use alternative means, beyond Web search, to continue it. In Information Retrieval, abandonment is often qualified as good abandonment (i.e., the information need has been fulfilled, the user is satisfied), or as bad abandonment (i.e., the information need stays unfulfilled, but the user still stops searching). The latter can happen for different reasons—lack of time, loss of interest, and frustration. In consequence, IR research revolves around understanding and predicting user’s decision to terminate the search (Diriye et al., 2012), to identify typical abandonment points during the information search process (Maxwell and Azzopardi, 2018) and to automatically determine indicators to identify good or bad abandonment for different search contexts (Williams et al., 2016; Williams and Zitouni, 2017).

Good or bad—the abandonment of a search might, however, not be a final decision. Complex tasks, in particular, can lead to search processes that are spread over multiple search sessions spanning a period of hours or even days. Louise, for instance, could, while writing her essay, realize that she is not yet able to explain the exact role of charged particles in thunderstorm creation and thus return to the search engine half an hour later. She could also ask her parent to read the essay before handing it in and then recommence the Web search based on the received feedback. A recent exploration of typical user behaviors related to multi-session searches was presented by Li et al. (2020). Findings include that, while searching on a multitude of topics, most study participants stated that their search intent was related to complex tasks, i.e., to higher levels of cognitive behaviors as classified in taxonomy of learning of Anderson and Krathwohl (2001).

In sum, research efforts toward detecting and qualifying abandonment and resuming of search exist, and there is investigation in complex user motivations and search intents. There are, however, some important gaps of research with respective to Search as Learning scenarios:

1. Can the present research on search abandonment be transferred to learning scenarios? Are there specific user behaviors that accompany learning-related search abandonment?
2. Can we develop mechanisms that support users in taking the correct decision concerning abandonment (i.e., scaffolding which allows the user to judge if sufficient material has been viewed)?
3. Are multi-session searches that relate to a learning intent distinguishable from those that are not related to learning?
4. How can a user be better supported to resume a learning session after abandonment for a certain time span?

#### Directions for Future Research

Existing studies rely on established interfaces and modes of interaction. This is reasonable for the present time but might be a limitation when trying to develop better support. While most users are confident in using standard results lists, alternative representations could offer more helpful support for the overall learning process. Examples in the literature include clustered result lists (Schrammel et al., 2009) or a grid presentation of the SERP (Kammerer and Gerjets, 2014) which provide better overviews on unknown domains.

Most of the current literature examines textual input and output for learning. However, multimedia resources are an essential component of Web information and a strong contributor to successful learning (Hoppe et al., 2018). Their role should be investigated more prominently.

In addition, current interfaces and interaction methods could be used to provide an improved learning experience if needed. In a first step, this demands for reliable detection of the user’s intention to perform a learning task. In a second step, the known search engine design could be gently extended by elements that proved helpful in general learning support systems. All while keeping the user within the familiar search environment. First SAL-oriented explorations propose introducing instructional scaffolding (Câmara et al., 2021), active reading tools (Roy et al., 2021), and assessments (Urgo and Arguello, 2022).

### Information Retrieval Backend (D)

**Figure 3:**
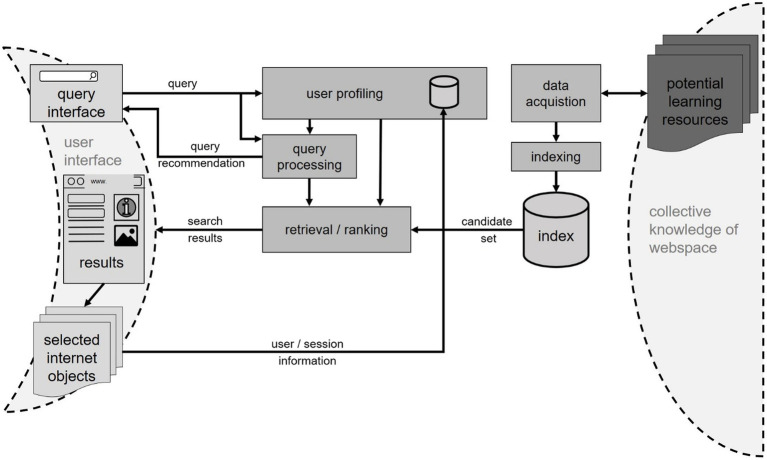
Information retrieval (IR) backend submodel with main components and connections to user interface and Web resources.

This section outlines the purpose and functionality of the fundamental components of an information retrieval backend for (web) search systems (Figure 3); for a comprehensive overview on Web information retrieval, refer to Croft et al. (2010). Following Kumar et al. (2017), the first component is the Web crawler: In order to provide Louise with a fast and responsive search engine, a sufficient amount of (Web) resources need to be collected long before she inputs her search query. Second, these resources need to be processed and made (machine) searchable by creating an *index*, allowing the system to retrieve results tailored toward Louise’s queries efficiently. After these preprocessing steps, the actual retrieval, ranking, or recommendation takes place. Here, a SAL-focused system would differ from common search engines, because their goal is to provide optimal results for an individual learning need. It can be assumed that optimal results for Louise might not be optimal for the next person. Thus, given Louise’s query, a subset of (Web) resources is compiled by comparing the query with document features in the index and thus, the SERP can be presented to her through the browser interface. This comparison is based on learning relevant metrics relevant to the individual learning preferences such as resource modality, -complexity, and -design rather than trends, popularity or paid advertisements. In parallel, different data sources are used to create or refine a user/learner profile enabling this learner-specific recommendation. A full discussion of approaches for the personalization of ranking and retrieval systems goes beyond the scope of this article. A good summary of current techniques can be found in Liu et al. (2020). The literature furthermore provides some examples that try to integrate search personalization into learning-oriented systems, e.g., Tan et al. (2012) integrate measurements of the Web resource’s comprehensibility for the individual user into the ranking mechanism; Liu and Jung (2021) seek to model the development of user interest, as a feature relevant specifically to learning-related tasks; Rieh et al. (2016) summarize how different kinds of search behavior can be linked to cognitive learning modes and thus, different search paradigms. Efforts of personalizing computer systems are, however, often met with critique as to their impact on information diversity (e.g., a recent reflection in Zanker et al., 2019 or study in Lai and Luczak-Roesch, 2019). Learning supporting search systems may collect and evaluate potentially sensitive data and should, therefore, be primed to use privacy-preserving mechanisms (El-Ansari et al., 2021).

#### Collecting Resources: Crawling

As Mirtaheri et al. (2014) state, a WebCrawler is software that starts from a set of seed URL(s) and downloads all Web pages that are associated with these URLs. From Louise’s perspective, this procedure is performed before the actual search session and cannot be influenced directly. Therefore, the operators of the search engine need to anticipate the types of resources visitors of their site are looking for. In regular Web crawling, the crawler parses the content of a seed page to discover new URLs that can be added to the set of seed URLs. This process continues iteratively until either the seed URL list is empty or a predefined number of crawled websites has been reached. *Focused Web crawling* aims at finding websites with either a given theme or topic (e.g., computer science, sports, and biology), a website type (e.g., forums, blogs), or a content type (images, videos). This behavior is defined by the crawler’s prioritization policy. In Louise’s case, depending on her search engine of choice, she (presumably unknowingly) expects the topic *natural sciences* to be covered. To predict whether a discovered URL fits the desired features *before* downloading it, a relevance score is computed based on features and patterns extracted from the URL and previous pages that linked to it (Mirtaheri et al., 2014). In this way, the order of the websites to be accessed by the crawler is determined.

These definitions assume that the entire content of a website is reachable through URLs. However, traditional Web crawlers cannot deal with the complexities posed by interactive websites that rely on user input to generate content. This happens whenever the Website is an interface to a database that relies on user input to retrieve content. The field of Deep Web crawling was recently born to address this issue. It models a website as a directed graph, and the World Wide Web can be understood as a very large set of such graphs. The goal of Web crawling is to discover all nodes in this set of graphs.

Madhusudan and Poonam (2017) discuss how the challenge of crawling the deep Web can be approached since it makes up approximately 96% of all Web content. Recently, Wang et al. (2019) proposed *SmartCrawl*, which is designed to maximize the return of hidden records from a database, given a set of queries and a fixed budget (e.g., number of API calls per day). Meschenmoser et al. (2016) outline and provide solutions for the common challenges when crawling scientific resources like pagination (splitting the results into pages), dynamic contents (page updates when scrolling to the bottom), and access barriers like obfuscated URL parameters and robot detection mechanisms. In conclusion, an advanced Web crawler is a requirement for an information retrieval backend that can provide comprehensive Web resources to learners like Louise. Due to the ever-growing amount of Web resources and irrelevant Web data, e.g., generated through advertisements or other unrelated information, current research methods focus on (a) the identification of websites worth crawling and (b) filtering based on their content.

#### Modeling Resources: Indexing

The purpose of indexing (web) resources in a search system is to enable retrieval and ranking algorithms (see Section “Retrieval and Ranking”) to *efficiently* find relevant resources given a user’s query. Normally, the vector space model (Manning et al., 2008) is used where the query and the Web resources are technically represented and described in feature vectors. The index must ensure that similarity between query and documents, e.g., using cosine similarity of vectors, in the collection can be computed in very short time. In an ideal world, a SAL-inspired indexing system would describe a website according to its content, including subtopics, provide information about the entry-level (beginners, intermediate, and experts), and give insights about the potential learning outcome for different types of users. As described in the Vignette, Louise wasted time watching a video that was not relevant to her query even though the title was fitting. An indexing technique that also analyses the content, rather than labeling a resource solely based on its title, would not have misled the ranking algorithm to consider it relevant.

Traditionally, a data structure called the *inverted index* was used to link a set of keywords to their associated documents (Manning et al., 2008). In their simplest design, these inverted lists are only able to determine if a token exists in a document but not their relevance to the user query. More advanced indexers, however, store additional information to support more complex search applications. A typical representation of text or other multimedia content relies on a *vector space model*. In this kind of IR model, text documents, images, etc., are represented through a (potentially high-dimensional) vector. The underlying idea is that similar text documents also share a similar vector representation in the vector space. A user’s query is then also converted to a corresponding vector of the same dimensionality. Consequently, the task of retrieving documents fitting the query is achieved by comparing the respective query vector with the vectors of the documents in the database (representing text documents, images, etc.). Also, a list can be sorted using similarity scores, where the more similar documents (compared with the query) are more likely to be retrieved. *Query-independent document scores*, such as click frequency, link popularity, URL size, and spam score are also used to ensure that more promising documents will be retrieved earlier (De Moura and Cristo, 2009). Depending on the amount of user information the search engine records, more results are shown to Louise that, for instance, people of her age preferred or that were clicked by other learners issuing similar queries.

The semantic indexing of multimedia content requires methods from computer vision, audio analysis and speech recognition, and natural language processing (Blanken et al., 2007). The challenge is to generate content-based features and indexes that go beyond user-generated tags, for instance, by analyzing image and video content through neural networks. Videos can be indexed as a whole but also based on the temporal segments, e.g., at the level of shots or scenes, to structure the content. However, the segmentation of educational videos poses a specific challenge since a topic transition does not necessarily evoke changes in shown content (e.g., in lectures) and vice versa. Attempts to solve this problem make use of speech transcripts and superimposed text (Tuna et al., 2015) or detection of slide changes (Jeong et al., 2015) to achieve a better performance.

These techniques enable modern retrieval systems to align user queries with particular video segments better and thus, provide learners like Louise with more relevant search results. While approaches from this research field are already capable of extracting semantically rich information from multimedia content, research with regard to the impact of multimodal features on knowledge gain has just started (Hoppe et al., 2018; Richter et al., 2018; Shi et al., 2019). Consequently, the full potential of multimedia content analysis has not been exploited for SAL scenarios. In the future, extensive user studies are needed to investigate how learning can be improved by automated resource analysis and indexing.

#### Query Processing

Once a search query is sent to the IR backend, stop words are removed, i.e., a set of commonly used words such as “the,” “is,” and “and,” they are considered as unimportant words for distinguishing the relevance of documents. In a next step spelling mistakes are corrected, which is for finding relevant items since the misspelled words or phrases are likely not included in the index. In the vignette, the IR backend assumes that “lighting” should be “lightning” and automatically corrects the supposed mistake.

Modern search engines use query expansion techniques to improve recall and precision or to personalize search results. More specifically, query expansion takes place in the backend: a set of new meaningful terms is added to a user’s original query to retrieve more relevant documents and reduce ambiguity (Azad and Deepak, 2019).

In SAL scenarios, the user is often unfamiliar with the search topic and may have difficulties formulating suitable queries. Whenever the learning goal is sophisticated, the user usually needs to issue more than one query to find adequate resources. An IR system may integrate query suggestion and query completion techniques to support the user in finding suitable queries. Query suggestion modules typically provide suggestions by guessing the user’s intention according to users’ past behavior (Ooi et al., 2015).

The output of the query processing module includes relevant queries that are sent to the query interface as suggestions to users and processed query and query expansion results that are passed on to the retrieval and ranking modules (Figure 3).

#### Retrieval and Ranking

The goal of the retrieval module is to find a set of Web resources that match the user’s search query, while the ranking module aims at ordering the resources according to the predicted relevance. Therefore, the similarity of the resource documents with regard to the query has to be measured using metrics like cosine similarity (e.g., Manning et al., 2008). Many different factors need to be considered when assessing the usefulness of a resource, for instance, relevance to the search query, quality, and suitability to the user. The ranked results are returned to the interface and displayed accordingly, e.g., in a vertical ranked list. A recent study could demonstrate that indeed different evaluation metrics are needed for different types of search intent (Liu and Yu, 2021).

To enable the aforementioned basic and advanced functions of the retrieval and ranking module, the input to the module typically includes features such as the processed queries, the user profile, the index of the resource collection, and other contextual information. Recent SAL research focuses, for instance, on the improvement of search result rankings for certain types of learning (e.g., Syed and Collins-Thompson, 2017). However, so far, studies on this direction are still rare; methods that can be generalized to different learning scenarios or can be embedded to real-world search engines are still missing.

#### User/Learner Profile

The user’s interactions with the interface create a consistent flow of information which then is used in the IR backend for query processing, retrieval, ranking, and the construction of a user or, more specific to our scenario, learner profile. Mojarad et al. (2018), for example, automatically identify groups of students with similar academic and behavioral characteristics. Modeling an SAL system after their example would, after continuous usage by Louise, enable it to determine whether she falls into one of six different types of learners: strugglers, average, sprinters, gritty, or coaster. This information can be leveraged further to determine the most suited type of educational resource, for example by modality, for her.

Generally, data used by the IR backend can be classified either as *explicit* or *implicit*. Explicitly collected user information depends on capturing the user’s personal information by collecting user feedback, for example, by filling in a form or by accessing personal information from other sources (think of Google Chrome being connected to one’s Google account). Data obtained this way may consist of demographic attributes such as the user’s name, address, telephone number, marriage status, job status, birthday, personal interest, and hobbies, or may include online transactions or Web activity. Implicit information is not directly gathered, but instead analyzed by intelligent data mining techniques that utilize user activity data (Kumbhare and Chobe, 2014). Implicit traces are created by analyzing all learner interface interactions, including login, software agents, enhanced proxy servers, cookies, and session IDs. Resources used for ranking have to be retrieved from the entirety of all the resources of the Internet. Since in SAL, those resources are to be used for learning, the IR backend has to select those suitable for the learner’s respective learning goals. By detecting a learner’s current information need, a SAL-oriented IR backend should be able to select appropriate resources, by means of, e.g., modality, complexity, length, and possibly source from an index constructed by Web crawling (see Section “Learner and Interface”). Studies as presented in Otto et al. (2021) that align learning outcome with the seen textual and visual content, are an attempt to gain these valuable insights. However, which of these properties are topic-independent a best fit for a given learner like Louise is not yet determined and topic of future research.

### Web Resources (D)

In the SAL context, Web resources are accessed by users through the IR system (see Figure 1). IR systems have to incorporate a sophisticated classification system to sort out the wheat from the chaff and select appropriate learning resources to support the user. The resources can be categorized as to whether they are used in a formal learning setting or not. Learning resources in formal learning settings include material created for educational purposes (e.g., textbooks, lecture slides) or other resources like video recordings from lectures in a classroom setting. Nowadays, more and more online activities are involved in formal education settings. In formal learning settings, we can also include online formal learning activities such as learning through course streaming, Massive Open Online Courses (MOOCs), Coursera, lecture videos in digital libraries, lecture videos in YouTube, SlideShare, etc. Learning resources outside formal learning settings include all other Web resources that are not generated for formal learning and can potentially also be used for intentional or non-intentional learning.

Various studies (Karanam et al., 2012; Van Oostendorp et al., 2012) have shown that the modality of learning resources influences the users’ resource selection, learning engagement, and learning outcome. Hence, it is necessary to consider the modality of the resource during the retrieval and ranking process. We classify the available resources in the Web space based on modality into the following categories:

- Text, e.g., textual content in Web resources.
- Still image in Web resources, e.g., the original file of format png, jpg, and bmp.
- Moving images, including animations and video.
- Audio information, e.g., podcasts, tutorials.

It is worth noting that a Web resource often combines different resource types or modalities, and it cannot always be simply assigned to a category. Furthermore, the assessment of the usefulness of a Web resource for learning is a complex task, different characteristics of the resource need to be taken into consideration:

- HTML structure of a website.
- Document complexity or difficulty.
- Linguistic characteristics and text styles (cf., Horne and Adali, 2017).
- Images [e.g., Syed and Collins-Thompson (2018) investigate the influence of images on user’s learning outcomes in web search].
- Other types of multimedia content.
- Quality of multimedia content.
- Relations between text and other media types.

Recently, empirical studies have begun to investigate relationships between domain-specific (Tang et al., 2021) as well as topic-independent (Yu et al., 2021) resource features and learning outcomes using supervised modeling techniques. Another line of research as addressed the specific importance of assessing characteristics of video-based resources to predict a knowledge gain from SAL-activities (Otto et al., 2021).

### Long-Term Dynamics

So far, we have focused primarily on single learning sessions or on a series of related learning processes that lead to data collection for a meaningful user profile. However, the Spaceship model also allows for the explanation of dynamics that happen on a larger time scale as a consequence of multiple learners using information technology repeatedly for different purposes. Such long-term dynamics have been laid out, for example, in the co-evolution model of learning and knowledge construction (Cress and Kimmerle, 2008; Kimmerle et al., 2015). Here, learning is conceptualized as a process that happens within an individual who tries to solve problems and make sense of the world. This individual can be understood as a cognitive system (Luhmann, 1984) that attempts to integrate information from its surrounding into a coherent meaning structure. On a larger timescale, continuous attempts at problem-solving lead to the continuous growth of knowledge on the side of the individual learner.

In turn, learners’ attempts to solve problems can also irritate the social system that surrounds them. For example, queries by a learner in a social media environment can create an irritation of the social system (Luhmann, 1984) because common beliefs and common knowledge of a community is questioned. If the community manages to resolve these irritations successfully and to restore a coherent meaning structure, this equals the creation of new knowledge within the knowledge community. Continuous irritations of the social systems through acts of individual learners lead to a continuous system drift in the form of the creation of more and more knowledge.

In the case of Louise, she learns from the videos by means of overcoming the friction between her beliefs about lightning and thunder and the information from the videos in a productive way. It is rather unlikely, albeit not impossible, that Louise stumbles upon something that will cause friction on the side of the knowledge community.

Within the Spaceship model, two other forms of system drifts can be described as well: The evolution of technology and the organization of knowledge resources within a community. The current state of user interfaces is already the result of more than two decades of human–computer interactions with digital information retrieval systems. We can also very well imagine that user interfaces will undergo severe changes over the upcoming years because of technological innovations that lead to certain changes in behavior patterns. An example for this is the increasing availability of virtual and augmented reality technologies. Those will enable very different ways of interacting with information in the near future.

**Figure 4:**
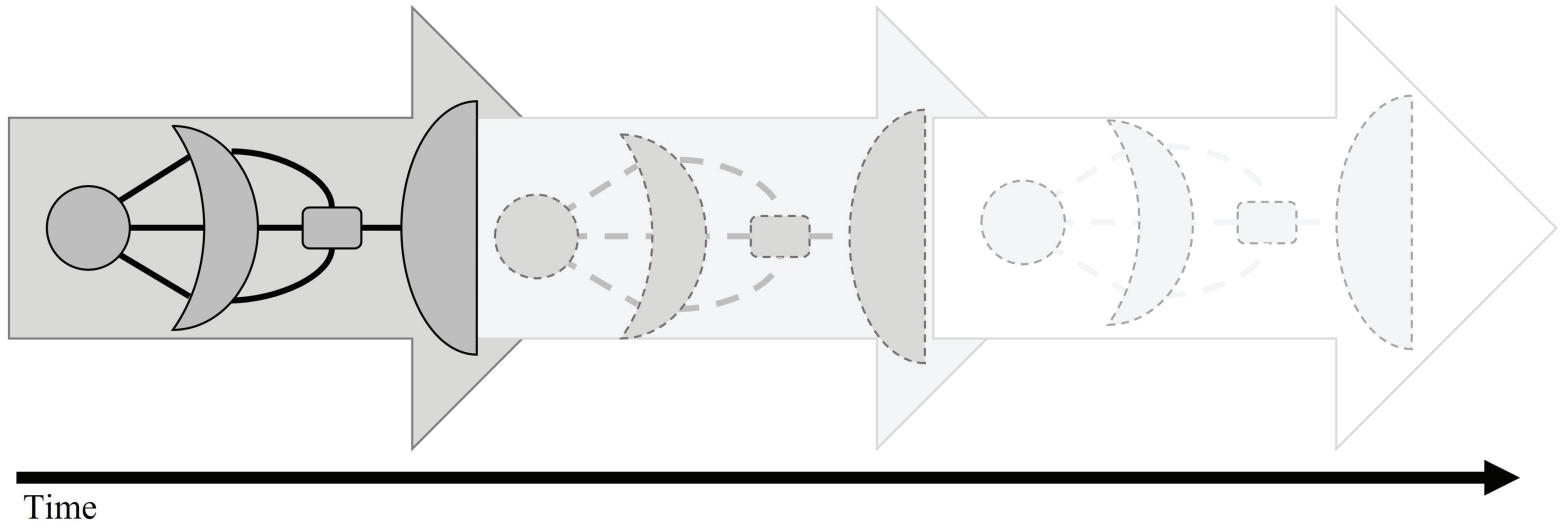
Evolution of the search as learning (SAL) spaceship over multiple user-environment interactions.

Also, on the level of the organization of the existing knowledge within a community, we can see continuous developments because of the learners’ interactions with digital (learning) environments. To provide a rather trivial example, certain events such as the large-scale outbreak of a disease can trigger certain information retrieval processes. Through collaborative filtering, these search processes will significantly affect the availability of certain pieces of information. In turn, the greater availability of this information can affect the way in which humans behave. Potential detrimental effects of certain types of recommender systems have been discussed over the last years, for example, under the umbrella terms filter bubbles (Pariser, 2011) and echo chambers (e.g., Quattrociocchi et al., 2016; Vakkari, 2016). Within filter bubbles, individual learners only receive a small and biased selection of the available knowledge resources on a given topic. This is a consequence of content-based recommendation systems recommending primarily resources similar to previously consumed resources; within echo chambers like-minded individuals share information *via* social media that reinforces their existing beliefs leading to eventual group polarization effects (Geschke et al., 2019). The introduced SAL Spaceship model offers a framework to conceptualize and to research the role of technology within such unwanted and undesirable developments. As a consequence of these system drifts which result from user-environment interactions, the whole spaceship in our model is constantly undergoing changes, although its rough shape remains more or less the same (see Figure 4; cf. Figure 1 in Kimmerle et al., 2015).

## Conclusion

In this paper, we have discussed the main aspects involved in search as learning activities from the perspective of both psychology and computer science. For this purpose, we have introduced our Spaceship model that offers orientation on the main factors in SAL, their connections and their dynamics. Our Spaceship model shows how intertwined technical and psychological processes are in SAL activities: On the one hand, learners have to adapt to a technical information environment to reach their learning goals; on the other hand, the technical environment should be designed as to adapt to the respective goals and preferences of the learner. At the moment, most research and many attempts at improving the fit between learners and information technology still address only one side of this system: They either address the learner’s behavior or the design of the information environment.

Open challenges and future directions, specifically from the computer science and information retrieval perspective, include the following:

– Evaluation protocols, datasets, and benchmarks. Performance evaluation of information retrieval systems and related metrics are geared toward measuring the relevance of a document to a given query. However, to aid SAL in search environments, retrieved resources—and eventually systems—have to be assessed with respect to their utility to a particular learning objective, i.e., a particular learning need within a specific context. This calls for novel evaluation protocols, metrics, and benchmarks to further facilitate progress in the field of SAL. A particular challenge in this context is the still very limited amount of experimentally obtained ground truth data able to capture both learning behavior as well as learning progress, i.e., knowledge gain.
– Generalizability and robustness. Prior SAL research has been focused on very specific use cases, for instance, learning types, disciplines, and topics. This has led to a plethora of work addressing SAL research questions in highly specific settings, where the generalizability of findings or machine learning models is not well understood.
– Application of SAL research for improved ranking and retrieval. Prior works are largely focused on understanding the user interactions in SAL settings and the interdependencies between search behavior, competence, knowledge gain, or learning objective with the goal to build predictive models able to classify in-session behavior. However, approaches toward actually exploiting such inferred information about learning objectives or user competence for improving ranking and retrieval in adaptive search settings are underinvestigated.
– Visualization and user interfaces for SAL. In addition, while it has been shown that the presentation of information to users has a significant impact on learning outcomes, search engines are still largely focused on presenting traditional SERPs to user without any regard to their learning object and actual intent. Future work should consider innovative means to present and contextualize information to aid efficient acquisition of knowledge.
– Multimodality. Whereas modality of resources is shown to have significant impact on learning outcomes, approaches that pay specific attention to the interplay between modality of resources, search behavior, and learning are still largely lacking and require further research.
– User profiling/customization. Whereas the consideration of user characteristics has already shown some promising results in experimental studies, much is still to be done in the field of user profiling and customization of search processes.
– Prevention of echo chambers and filter bubbles. In view of their immense cultural and political importance, many psychological and sociological studies have addressed over the last years unwanted results of information search processes such as the possible formation of echo chambers and filter bubbles. Still work on the application of findings from these studies on information search environments is still in its early stages.

It is imperative for researchers and practitioners in the SAL field to widen their horizon: In the case of psychologists, educators, and social scientists, it is necessary to obtain a thorough level of understanding of the technical infrastructure, whereas computer scientists and software architects might benefit from a thorough understanding of the psychological processes taking place in a learner. In both cases, it is also essential that the respective knowledge is updated on a regular basis: Just as technology advances rapidly, usage patterns and cognitive processes on the side of the users are also evolving as a consequence of adaptation processes (Firth et al., 2019).

So far, our model focuses on cases where a single learner engages in a learning activity. In the future, the Spaceship model could be expanded toward integrating both social interactions between learners and the belonging of learners into social groups.

## Author Contributions

JH, PH, AH, YK, CO, GP, MR, and RY wrote sections of the manuscript. JH, PH, RE, and SD contributed to conception and design of the manuscript. All authors contributed to the article and approved the submitted version.

## Funding

This work is financially supported by the Leibniz Association, Germany [Leibniz Competition 2018, funding line “Collaborative Excellence,” project SALIENT (K68/2017)].

## Conflict of Interest

The authors declare that the research was conducted in the absence of any commercial or financial relationships that could be construed as a potential conflict of interest.

## Publisher’s Note

All claims expressed in this article are solely those of the authors and do not necessarily represent those of their affiliated organizations, or those of the publisher, the editors and the reviewers. Any product that may be evaluated in this article, or claim that may be made by its manufacturer, is not guaranteed or endorsed by the publisher.
